# A novel biochip-based liquid biopsy for extracellular vesicle RNA detection in prostate cancer

**DOI:** 10.1080/15384047.2025.2593744

**Published:** 2025-12-03

**Authors:** Yanjun Diao, Ani Nan, Rui Li, Ting Ding, Zhuo Li, Juan Wang, Bingbing Zhu, Jinjie Li, Liu Yang, Lei Zhou, Jiayun Liu, Xiaoke Hao

**Affiliations:** a Department of Clinical Laboratory Medicine, Xijing Hospital, Air Force Medical University, Xi’an, Shaanxi, People's Republic of China; b Neonatal Disease Screen Center of Xi'an Maternity and Child Healthcare Hospital, Xi’an, Shaanxi, People's Republic of China; c Department of Laboratory Medicine, the First Affiliated Hospital of Xi’an Medical University, Xi’an, Shaanxi, People's Republic of China

**Keywords:** Prostate cancer, Extracellular vesicles, RNA biomarkers, Liquid biopsy, Biochip-based detection

## Abstract

**Background:**

Prostate cancer (PCa) is a major health concern, and current PSA screening is limited by low specificity and the risk of overdiagnosis. Extracellular vesicle (EV)-derived RNA biomarkers offer a promising non-invasive alternative for early detection.

**Methods:**

We utilized a tethered cationic lipoplex nanoparticle (TCLN) biochip for amplification-free EV RNA detection at the single-vesicle level. Eight candidate RNAs (four miRNAs, three mRNAs, and one lncRNA) were profiled in serum and urine samples from PCa patients, benign prostatic hyperplasia (BPH) patients, and healthy controls (HC). Diagnostic and risk stratification performance was evaluated in discovery and validation cohorts, with qRT-PCR used for validation.

**Results:**

TCLN reliably detected the candidate RNA biomarkers from PCa cell lines and clinical samples, with strong concordance to qRT-PCR. Serum EV RNAs (miR−141, miR−375, Let-7c) and urine EV RNAs (miR−141, miR−375, PCA3 lncRNA, T1-E2) effectively distinguished PCa patients from controls. Combined EV RNA biomarkers in serum and urine achieved diagnostic area-under-the-curve (AUCs) of 0.824 and 0.741, respectively, surpassing those of prostate-specific antigen (PSA) alone. Serum miR−141, miR−375, and urine miR−141, miR−375, and PCA3 lncRNA, also showed remarkable correlations with PCa Gleason score (GS), tumor stage, and metastatic status.

**Conclusion:**

The TCLN biochip enables sensitive, amplification-free detection of EV RNA biomarkers from serum and urine. Key markers such as miR−141, miR−375, and PCA3 showed strong diagnostic and risk stratification value in PCa. This non-invasive approach holds promise for improving early detection and clinical risk assessment.

## Introduction

Prostate cancer (PCa) is one of the most prevalent malignancies in men worldwide and a leading cause of cancer-related morbidity and mortality.^1^^,^^2^ According to the GLOBOCAN 2020 database, PCa ranks as the second most diagnosed cancer and the fifth leading cause of cancer death among men globally.^3^ In clinical practice, the prostate-specific antigen (PSA) test remains the most widely used biomarker for PCa screening.^4^^,^^5^ However, PSA fails to provide sufficient specificity, often resulting in false positives and unnecessary biopsies, which limits its utility in risk stratification and PCa early detection.^2^^,^^4^^,^^6^ Therefore, there is an urgent need for novel biomarkers with improved sensitivity and specificity to enable both early diagnosis and accurate assessment of PCa malignancy and progression.

Extracellular vesicles (EVs) are membrane-bound lipid vesicles secreted by most cell types, including tumour cells^7^. EVs carry a rich cargo of proteins, lipids, DNA,^8^ and various RNA species, such as microRNAs (miRNAs), long non-coding RNAs (lncRNAs), and mRNA fragments,^9^ many of which may be cancer-specific, making them attractive candidates for liquid biopsy-based cancer diagnostics.^10^^,^^11^ In PCa, EV-associated RNAs (EV RNAs) have demonstrated promising diagnostic and prognostic potential.^12-22^ For example, EV-derived miR−141 and miR−375 are consistently upregulated in PCa patients and correlated with disease status.^23^^,^^24^ Other miRNAs (miR-19b-3p, miR−101-3p) and lncRNAs (PCA3, SAP30L-AS1, SChLAP1) have also been recognised for their diagnostic and prognostic relevance.^25-28^ Importantly, EVs can be readily isolated from minimally invasive or non-invasive biofluids, such as serum and urine,^29^ enabling accessible and repeatable molecular testing. Compared to free-circulating RNAs, EV RNAs may offer better stability and specificity, highlighting their value as clinically relevant biomarkers. Emerging evidence suggests that EVs protect their RNA cargo from enzymatic degradation in circulation, thereby providing greater long-term stability.^30-33^ In addition, EV-associated RNAs are thought to be selectively packaged, reflecting the molecular characteristics of their releasing cells more faithfully than fragmented, freely circulating RNAs.^34^ These merits render EV RNAs ideal candidates for non-invasive cancer diagnostics.

Despite their great clinical potential, conventional sequencing-based detection methods of EV RNA face technical limitations, including low sensitivity, non-specific background signals, and time-consuming protocols.^35^^,^^36^ To overcome these challenges, a nanotechnology platform known as the Tethered Cationic Lipoplex Nanoparticle (TCLN) biochip has been developed.^37-41^ Specifically, this platform utilises positively charged cationic lipid nanoparticles tethered to a gold-coated chip surface to electrostatically enrich negatively charged EVs from biofluid samples. Although this charge-based enrichment can broadly capture negatively charged vesicles, subsequent hybridisation with fluorescently labelled molecular beacons (MBs) confers high target specificity. The MBs are designed as stem-loop oligonucleotide probes with a fluorophore and quencher that remain quenched in the closed hairpin conformation; upon hybridisation with complementary EV RNA targets, the MBs open, separating the fluorophore from the quencher and producing a fluorescence signal. The fluorescence is then quantified by total internal reflection fluorescence microscopy (TIRFM), which excites fluorophores within a ~300 nm evanescent field above the surface, helping to further filter out background signals from larger or non-vesicular contaminants. With these merits, TCLN enables rapid, amplification-free detection of low-abundance RNAs in small-volume serum or urine samples, showing great promise in the early diagnosis of lung cancer.^38-40^ Specifically, TCLN achieved an area-under-the-curve (AUC) of 0.970 in distinguishing early-stage non-small cell lung cancer (NSCLC) patients from normal controls, far surpassing the performance of qRT-PCR.^40^ TCLN has also demonstrated effective identification of lung cancer patients with gefitinib resistance.^39^ However, its potential clinical application in prostate cancer has not yet been explored.

In this study, we demonstrate the use of TCLN technology for EV RNA profiling in prostate cancer patients, assessing its diagnostic performance and risk stratification value. Specifically, we assessed a panel of eight EV RNAs, including miRNAs, mRNAs, and lncRNAs, in both serum and urine samples from PCa patients, benign prostatic hyperplasia (BPH) patients, and healthy controls (HC). These eight targets were chosen based on their strong prior evidence of diagnostic relevance in prostate cancer, with the goal of maximising diagnostic performance in this initial translational feasibility study, while also taking into account sample volume limitations. Beyond distinguishing cancer from non-cancer cases, we also explored whether specific EV RNAs could reflect PCa progression, including Gleason score (GS), tumour stage, and metastatic status.

## Materials and methods

### Patients and sample collection

Patients were recruited at the Department of Laboratory Medicine, First Affiliated Hospital of Air Force Medical University between March 30, 2022 and June 30, 2024. Inclusion criteria included (1) patients diagnosed with PCa and/or BPH by histology; and (2) no prior treatment and no historical or concurrent malignancies. Age- and sex-matched healthy individuals with no clinical or pathological evidence of PCa or other malignancies were recruited from health examination centres and included in the HC group. Blood and urine samples were collected from a training cohort of PCa patients (126 serum and 135 urine samples) and a follow-up validation cohort (124 serum and 124 urine samples) (Table 1). Clinical and biochemical data, including age, serum PSA levels, Gleason grade, clinical tumour stage, and metastasis status (derived from TNM staging at the time of sample collection), were systematically recorded. Informed written consent was obtained from all participants. The study was approved by the medical ethics committee of the First Affiliated Hospital of Air Force Medical University (Approval KY20223465−1).

**Table 1.** Clinical characteristics of participants (serum and urine samples).

Sample for RT-PCR testing										
Characteristic	Serum					Urine				
	HC	BPH	PCa	p		HC	BPH	PCa	p	
				PCa vs HC	PCa vs BPH				PCa vs HC	PCa vs BPH
Total number	48	60	63	/	/	45	57	69	/	/
Age (years) median (IQR)	66.0 (64.5−70.0)	64.0 (62.0−78.0)	69.0 (59.0−77.0)	ns	ns	63.3 (59.0−72.1)	65.4 (63.1−79.0)	62.7 (61.5−66.3)	ns	ns
PSA (ng/mL) median (IQR)	1.1 (0.6−1.6)	8.8 (4.6−14.5)	40.5 (5.9−581.7)	*	*	1.78 (076−1.99)	21.32 (9.75−33.67)	58.82 (16.38−76.45)	*	*

			Sample for TCLN testing (Training cohort)							
Characteristic	Serum					Urine				
	HC	BPH	PCa	p		HC	BPH	PCa	p	
				PCa vs HC	PCa vs BPH				PCa vs HC	PCa vs BPH
Total number	126	126	126	/	/	135	135	135	/	/
Age (years) median (IQR)	63.0 (64.5−70.0)	66.0 (62.0−78.0)	67.0 (59.0−77.0)	ns	ns	66.0 (59.0−77.0)	65.0 (62.0−78.0)	66.5 (64.5−70.0)	ns	ns
PSA (ng/mL) median (IQR)	1.12 (0.61−1.63)	8.82 (4.22−11.85)	39.51 (6.95−92.70)	*	*	1.54 (0.36−1.82)	18.45 (2.79−23.62)	65.31 (6.52−83.04)	**	*

			Sample for TCLN testing (Validation cohort)							
Characteristic	Serum					Urine				
	HC	BPH	PCa	p		HC	BPH	PCa	p	
				PCa vs HC	PCa vs BPH				PCa vs HC	PCa vs BPH
Total number	124	124	124	/	/	124	124	124	/	/
Age (years) median (IQR)	68.0 (59.0−78.0)	65.0 (62.5−77.0)	66.0 (64.0−71.0)	ns	ns	64.0 (61.0−72.0)	69.0 (67.5−81.0)	67.0 (64.0−72.0)	ns	ns
PSA (ng/mL) median (IQR)	1.76 (0.31−1.80)	11.64 (1.39−11.25)	53.35 (2.52−52.56)	**	*	1.34 (0.31−1.48)	27.76 (1.39−11.60)	85.61 (2.52−53.04)	*	*

### Cell line verification

The four cell lines used in this study (PC3, RRID:CVCL_0035; LNCaP, RRID:CVCL_0395; DU145, RRID:CVCL_0105; RWPE−1, RRID:CVCL_3791) were purchased from the Cell Resource Centre of the Shanghai Institute of Life Sciences, Chinese Academy of Sciences. To ensure the identity and purity of the cell lines, all cell lines were authenticated using short tandem repeat (STR) analysis, and the results showed that their STR profiles completely matched the reference data from the ATCC, DSMZ, and JCRB databases, with no cross-contamination detected. Additionally, all cell lines were tested for mycoplasma contamination using the MycoAlert™ Mycoplasma Detection Kit (Lonza), and the results were negative. All cell lines were authenticated prior to experiments and re-validated every three months during culture to ensure their identity and purity. The cell lines used in the experiments did not exceed 20 passages.

### Exosome isolation and characterisation

EVs were isolated using specimen-specific protocols and no freeze-thaw cycles or long-term storage were applied prior to EV isolation and analysis. For cell culture supernatant, EVs were separated using differential centrifugation. The initial centrifugation was performed at 2,000× g for 10 minutes, followed by two rounds of high-speed centrifugation at 120,000× g for 70 minutes using a Beckman Coulter Type 45 Ti rotor. A total of 10 mL of cell culture supernatant was used for each sample. For serum samples, EVs were isolated using Exosupur® columns (Echobiotech, China). The serum was first centrifuged at 300× g for 10 minutes, followed by another spin at 2,000× g for 10 minutes. A total of 200 μL of the resulting supernatant was then directly processed for purification using the extractor, followed by re-suspension in phosphate-buffered saline (PBS), resulting in a final volume of 500 µL. For urine samples, midstream first-morning urine was collected from participants who had not undergone a prostate rectal exam or massage. For each sample, a total of 15 mL of urine was centrifuged at 10,000× g for 30 minutes to remove cell debris and sediment. For urine-derived EVs isolated through ultrafiltration combined with precipitation, the samples were first centrifuged sequentially at 300× g for 10 minutes and 2,000× g for 10 minutes before undergoing ultrafiltration with a 100 kDa membrane. The ultrafiltrate was then concentrated to 1/10 of its original volume, after which PEG precipitant (System Biosciences, Mountain View, CA, USA) was added. The isolated EVs were finally resuspended with PBS, resulting in a total volume of approximately 500 µL for each sample. The sample was incubated at 4 °C for 2 hours before testing. This processing protocol has been reported to effectively reduce uromodulin interference.^42^

Transmission electron microscopy (TEM) (Tecnai, USA) was used to examine EV morphology. Nanoparticle tracking analysis (NTA) using a ZetaView instrument (Particle Metrix, Germany) was performed to determine EV size distribution and particle concentration.

### Screening of candidate EV RNA biomarkers for PCa

With a focus on near-term clinical value, we employed a targeted selection approach in screening the panel of EV RNA biomarkers based on the following criteria: 1) consistent reports of upregulation or dysregulation in prostate cancer across multiple independent studies, 2) demonstrated detectability in extracellular vesicles from biofluids, and 3) evidence of potential clinical relevance in terms of diagnostic value. We selected a total of eight EV RNA biomarkers, including miRNAs, lncRNAs, and mRNA fusion transcripts, for evaluation in PCa. Among the miRNAs, miR−141 and miR−375 have been consistently reported to be elevated in PCa and associated with disease progression^12^^,^^43^. Additional candidates such as miR−21, miR-19b, and miR−574-3p have also been implicated in prostate tumorigenesis and found in EVs from urine or serum.^12^^,^^44-47^ In contrast, Let-7c is a tumour suppressor downregulated in PCa.^48^ In the lncRNA category, PCA3 is one of the most widely studied non-coding RNAs in PCa diagnostics, particularly in urinary EVs.^26^ We also included SAP30L-AS1 and SChLAP1, both of which are overexpressed in aggressive PCa and have been detected in circulating EVs.^27^^,^^49^ For mRNA biomarkers, we included the transcript mRNA of PSA, the most common upregulated biomarker of PCa.^5^ We also targeted the TMPRSS2:ERG fusion transcript, which occurs in approximately 50% of PCa cases and is considered a hallmark genetic alteration.^50^^,^^51^ Since the most common fusion isoforms involve a junction between TMPRSS2 exon 1 and ERG exon 2 or exon 4, two MB probes were designed to detect the corresponding fusion transcripts (T1-E2 and T1-E4).

The sequences of the eight candidate biomarkers and their corresponding MB used for TCLN detection are listed in Table 2. After synthesis, all eight MBs were encapsulated in cationic lipid nanoparticles and immobilised onto a 24-well biochip. This setup enables the electrostatic capture of negatively charged EVs, leading to the formation of larger nanoscale complexes.

**Table 2.** Eight candidate EV RNA targets and their corresponding molecular beacon sequences.

Type	Name		Accession number in database	Sequence of RNA	Sequence of MB
4 miRNA	hsa-miR−141-3p		miRBase: MIMAT0000434	5'-UGGAGUGUUGUAAUGGUUUGUG−3'	5'-FAM-CTGGAACAGGATTGGTGGAGACATGGAAGGTCAGGT−3'BHQ−1
	hsa-miR−375-3p		miRBase: MIMAT0000473	5'-UGUUUGUUUUGUUGUUGUUGUG−3'	5'-FAM-CTGGAAGGGAGGTGTTTGTGAGGTTGGTGAGGAGGAGGAGAG−3'BHQ−1
	hsa-miR−21-5p		miRBase: MIMAT0000076	5'-UAGCUUAUCAGACUGAUGUUGA−3'	5'-FAM-CTGGAAGAGGAGGAGGAGAGGUAGCUUAUCAGACUGAUGUUGA−3'BHQ−1
	hsa-let-7c-5p		miRBase: MIMAT0000434	5'-FAM-CTGGAAGAGGAGGAGGAGAGGUGAGGUAGUAGGUUGUAUGUUU−3'BHQ−1	5'-FAM-CTGGAAGAGGAGGAGGAGAGGUGAGGUAGUAGGUUGUAUGUUU−3'BHQ−1
3 mRNA	PSA		NCBI: NG_012858.1	5'-GCGCTGAGAGGAAATGAGAGGAAGGAGGAGGAGGAGGAAGAGGAAGAGGAAGGAAGGGAAGG−3' (Exon 3)	5'-FAM-CTGGAAGAGGAGGAGGAGAGGAAGGAAAGGAAGGAAGGAGGAAGGAGGAGGA−3'BHQ−1
	TMPRSS2:ERG fusion gene	T1-E2	NCBI: NM_001195004.1	5'-ATCGGTAGCAGGATTAGGAGAGGAGTGCAGTGAG−3'(sequence of the fusion region between Exon T1 and Exon E2.)	5'-FAM-CTGGAAGAGGAGGAGGAGAGGAGTAGCAGGATTAGGAGAGGAGTGCAGTGAG−3'BHQ−1
		T1-E4		5'-ATCGGTAGCAGGATTAGGAGAGGAGAGTGCAGTGGAG−3'(sequence of the fusion region between Exon T1 and Exon E4.)	5'-FAM-CTGGAAGAGGAGGAGGAGAGGAGTAGCAGGATTAGGAGAGGAGTGCAGTGGAG−3'BHQ−1
1 lncRNA	PCA3		NCBI: NR_132312.2	5'-GGTAGGAGGAGAGGAGGGAGGAAGGAAGGAGGAAGGAAGGAGGAGGAGGAGGAGGA−3'(Exon 1)	5'-FAM-AGGAAGGAAGGAAGGAGGAAGGAAGGAAGGAAGGAGGAGGAAGGAGGAAGGAGGA−3'BHQ−1

### EV RNA isolation and validation using qRT-PCR

EVs were isolated from equal volumes of serum (400 µL) or urine (20 mL) samples collected from HC, BPH, and PCa patients. To minimise variability caused by differences in EV yield, all samples were standardised to 1× 10^9^ particles/100 µL (10^10^ particles/mL) as measured by NTA prior to RNA extraction. For samples below this concentration, the actual particle count was recorded and used to apply a normalisation factor during data processing (Normalised expression = original 2^−Ct× [10^10^/actual particle number]). Exogenous spike-in RNAs were added immediately after EV isolation and before RNA extraction to ensure consistent normalisation across samples. For short RNAs (e.g., miR−141, miR−375), cel-miR−39 (Thermo Fisher, Cat. No. A25576, length 22 nt) was used as the normalisation reference, and for long RNAs (mRNA and lncRNA targets, e.g., PSA, TMPRSS2:ERG, PCA3), ERCC−00096 (Thermo Fisher ERCC RNA Spike-In Mix, Cat. No. 4456740; length ~1060 nt) was used.

Total RNA was extracted using the miRNeasy Serum/Plasma Kit (Qiagen, Hilden, Germany) following the manufacturer’s protocol, yielding a final elution volume of 15 µL. Quantitative reverse transcription polymerase chain reaction (qRT-PCR) was performed using the PrimeScript RT Reagent Kit and SYBR Premix Ex Taq Kit (Takara Bio, Inc., Shiga, Japan). The reverse transcription reaction was carried out in a 10 µL mixture containing 2 µL of 5× PrimeScript buffer, 0.5 µL of PrimeScript RT enzyme mix, 0.5 µL of a gene-specific primer, and 7 µL of RNA extract. The reaction was incubated at 37 °C for 15 minutes, followed by 85 °C for 5 seconds, and then maintained at 4 °C. Next, 2 µL of complementary DNA (cDNA) was amplified in a 20 µL qPCR reaction mixture, consisting of 10 µL of 2× SYBR Premix Ex Taq, 0.8 µL of gene-specific primers, 0.4 µL of 50× ROX Reference Dye II, and 6.8 µL of nuclease-free water. qRT-PCR was conducted on an ABI 7500 Fast Detection System (Applied Biosystems, Foster City, CA, USA) under the following cycling conditions: an initial denaturation at 95 °C for 30 seconds, followed by 40 cycles of 95 °C for 3 seconds and 60 °C for 30 seconds. At the end of the PCR cycles, melting curve analysis was performed to verify the specificity of the PCR products. All reactions were conducted in triplicates. The bulge-loop qRT-PCR primers for the candidate EV RNA biomarkers were sourced from RiboBio. The relative expression levels were calculated using the comparative 2^−ΔΔCt method, with ΔCt values normalised to the corresponding spike-in RNA control for each RNA class.

### Detection of PCa EV RNA biomarkers using the TCLN assay

All clinical serum and urine samples were processed promptly after collection to ensure EV integrity and minimise degradation. No freeze-thaw cycles or long-term storage were applied prior to EV isolation and analysis. TCLN biochips were obtained from Hangzhou Dixiang Biotech, and EV RNA detection using the TCLN assay was performed following previously published protocols.^40^ In brief, TCLN biochips were fabricated by depositing a 15 nm thin gold (Au) layer onto a glass coverslip using a Denton e-beam evaporator (DV-502A, Denton Vacuum, NJ, USA). A mixed thiol self-assembled monolayer (SAM) was then formed on the gold surface, serving as an anchoring membrane.

**Figure 1.**
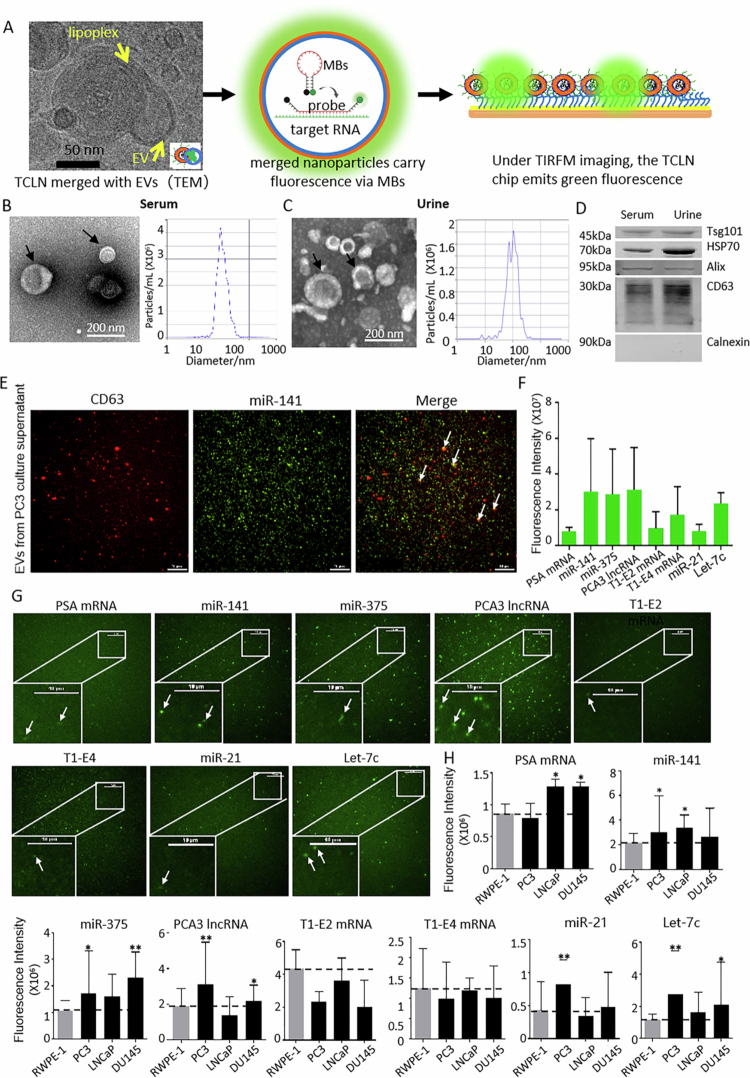
Characterisation of EVs Derived from Clinical Samples and Preliminary Assessment of TCLN-Based Detection of Candidate EV RNA Biomarkers for prostate cancer (PCa). (A) A schematic representation of the TCLN-based detection method for EV RNA biomarkers. (B-C) TEM images of EVs isolated from serum (B) and urine samples (C) of PCa patients show uniform distribution, consistent morphology, and predominantly cup-shaped or spherical structures. NTA analysis confirms that EV diameters typically range from 30 to 150 nm. (D) Western blot analysis detects EV markers (TSG101, HSP70, CD63, Alix) in both serum- and urine-derived EVs. The absence of Calnexin indicates minimal cellular contamination. (E) TIRFM analysis confirms the co-localisation of miR−141 with EVs derived from PC3 cell culture supernatant. CD63-labelled EVs emit red fluorescence, while miR−141 fluoresces green upon hybridisation with molecular beacon (MB) probes. The resulting orange fluorescence signals, indicated by arrows, confirm miR−141 encapsulation within EVs. (F) Mean fluorescence intensity ± standard deviation (SD) for each biomarker. (G) Representative TCLN images display eight candidate RNA biomarkers detected in EVs from PC3 cell culture supernatant. White boxes indicate selected regions of interest, which are shown at higher magnification to visualise individual EVs emitting RNA-specific fluorescence signals (highlighted by arrows). Scale bars represent 10 μm. (H) Levels of the EV RNA candidates in different cell lines. The data have been normalised relative to the fluorescent intensity of RWPE−1 EVs. All values are presented as the mean ± SD of the fluorescence intensity (*p <0.05, **p <0.01, ***p <0.001; *n* = 100 images).

TCLNs containing MB for the eight candidate EV RNA biomarkers (synthesised by Sigma-Aldrich, MO, USA) were immobilised onto the glass slide surface via a biotin-avidin linkage. Each MB was fluorescently labelled with Fluorescein Amidite (FAM). EVs carrying target RNA biomarkers merged with TCLN nanoparticles, allowing the MBs to hybridise with their complementary RNA sequences, resulting in fluorescence emission. A TIRF microscope was used to visualise and quantify RNA expression in the EVs (Figure 1A). Specifically, TCLN detection was performed using a Nikon Eclipse Ti-E total internal reflection fluorescence microscope (Nikon Instruments Inc.), equipped with an Andor iXon EMCCD camera and a 100× oil-immersion objective. The exposure time was set to 200 ms, and the imaging depth was controlled to <300 nm. A total of 100 randomly distributed field-of-views (FOVs) were obtained for each sample. Image acquisition and analysis were conducted using NIS-Elements software and a custom MATLAB script optimised for thresholding and background subtraction. Specifically, a lower threshold was established based on PBS blank controls to eliminate non-specific background signals generated during imaging. This threshold serves as a system noise floor, ensuring that only signals above baseline fluctuations are considered. All images were processed based on this cutoff value and only signals with intensities above this cutoff were retained for downstream analysis. A binary mask was applied to remove background noise, followed by measurement of the average fluorescence intensity for each image. The mean of the average fluorescence intensity of all FOVs was calculated for each sample as the readout of a well.

### Data processing and statistical analysis

Each sample was tested independently at least three times, and the final result for each sample was expressed as the mean fluorescence intensity and was included for analysis. For comparisons between TCLN and qRT-PCR results, individual fluorescence intensities or Ct values were normalised to the mean value of the HC group to calculate fold changes, enabling consistent trend evaluation across the two platforms. Statistical analyses were conducted using SPSS 26.0 (IBM Corp., NY, USA) and GraphPad Prism 7 (GraphPad Software Inc., CA, USA). Statistical significance was determined using Student’s t-test (unpaired t-test), chi-square test, and Fisher’s exact test, as appropriate.

Multivariable logistic regression modelling was applied to assess the combined diagnostic value of multiple EV RNA biomarkers. For each biomarker panel, a predictive equation was constructed by

$$\mathrm{logit}\left(P\right)=\beta_{0}+\sum_{i=1}^{n}\beta_{i}*{\mathrm{Marker}}_{i},$$

where P represents the predicted probability of PCa, and $\beta_{1}$~$\beta_{n}$ are the regression coefficients estimated from the training cohort for each marker. Receiver operating characteristic (ROC) curves were generated by plotting the true positive rate (sensitivity) against the false positive rate (1-specificity). The AUC values were calculated along with their 95% confidence intervals (95% CI). Cutoff values were established based on the point on the ROC curve that corresponded to the maximum Youden’s index (YI).^52^ All statistical tests were two-sided, and a *p*-value of <0.05 was considered statistically significant.

## Results

### Preliminary evaluation of TCLN-based detection of candidate RNA biomarkers in EVs from cell culture supernatant

Serum- and urine-derived EVs were isolated and analysed for size, morphology, and molecular markers using TEM, NTA, and Western blotting. The isolated EVs were validated by TEM imaging showing cup-shaped morphology (Figure 1B,C), NTA analysis demonstrating a size distribution predominantly between 30–150 nm, and Western blot confirming expression of canonical EV markers (TSG101, HSP70, CD63, Alix) and absence of Calnexin (Figure 1D). These data indicate successful isolation of EVs with minimal cellular contamination.

To assess the effectiveness of TCLN technology in detecting RNA biomarkers in PCa-derived EVs, EVs were isolated from the PC3 metastatic prostate cancer cell line and analysed for the co-localisation of miR−141 with CD63-labelled EVs. miR−141 is one of the most well-documented PCa-associated miRNAs, and our previous studies have confirmed its elevated expression in PC3-derived EVs.^16^^,^^53^ TCLN-based imaging revealed that a subset of CD63-positive EVs contained miR−141. However, not all CD63-positive EVs exhibited miR−141 signals (Figure 1E), suggesting that miR−141 is not uniformly distributed across all CD63-expressing EVs. Additionally, since only an estimated 20%-50% of EVs express CD63,^54^ many EVs in the population may contain miR−141 but lack CD63 expression.

Additionally, TCLN technology was used to evaluate the expression levels of the eight candidate RNA biomarkers. The results confirmed successful detection of all eight markers, with miR−141, miR−375, PCA3 lncRNA, and Let-7c showing relatively higher expression levels and clearer imaging signals compared to the rest of the candidates (PSA mRNA, T1-E2 mRNA, miR−21, and Let-7c) (Figure 1F,G).

We then analysed the expression of the eight candidate RNA markers in the non-cancerous RWPE−1 cell line and three prostate cancer cell lines, including PC3, LNCaP, and DU145, using TCLN and TIRF microscopy (Figure 1H). The data showed that fluorescent signals for miR−141, miR−375, PCA3 lncRNA, miR−21, and Let-7c were 1.3 to 2 times higher in PC3 cells compared to RWPE−1, with PC3 showing greater expression than DU145. In contrast, DU145 showed higher levels of PSA mRNA and miR−375 compared to PC3. Additionally, PSA mRNA and miR−141 expression were elevated in LNCaP cells compared to RWPE−1. However, T1-E1 and T1-E4 mRNA did not exhibit significant increase in any of the three cancer cell lines compared to RWPE−1.

### Validation of candidate biomarkers using qRT-PCR in a small clinical sample cohort

**Figure 2.**
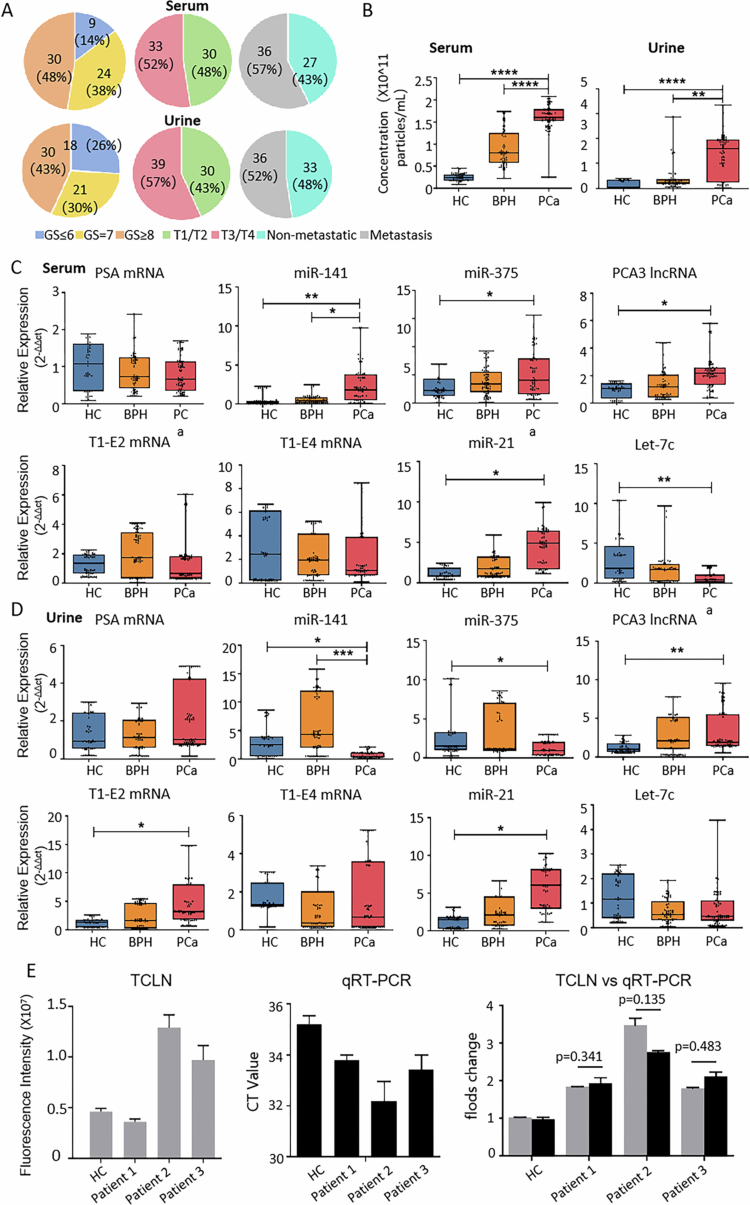
RT-PCR Validation of Candidate EV Biomarker Expression in Clinical Samples with Limited Sample Size. (A) Gleason scores, tumour staging, and metastatic status of patients in the PCa group (63 serum and 69 urine samples). (B) Exosome concentrations in the clinical samples measured by NTA analysis. (C) and (D) RT-PCR analysis of candidate EV RNA biomarker expressions in serum samples from healthy controls (HC), benign prostatic hyperplasia (BPH), and PCa patients. Sample sizes are detailed in Table 1. Boxplots represent the interquartile range (IQR) across different groups. The horizontal lines and whiskers represent the median and the maximum and minimum values, respectively. Student’s t-test was used to assess statistical differences between groups (*p <0.05, **p <0.01, **p <0.001). (E) TCLN and qRT-PCR-based miR−141 detection in EVs from serum samples of normal controls and three PCa patients show consistent trends.

To confirm the reliability of the eight candidate RNA biomarkers identified in the literature, we first performed qRT-PCR validation before implementing TCLN detection. Due to the limited sample volume, we selected a small cohort of PCa patients, including 63 serum samples and 69 urine samples, for qRT-PCR validation (Figure 2A). A total of 48 HC and 60 BPH serum samples, as well as 45 and 57 urine samples, were selected for comparison.

Following EV isolation from these clinical samples, NTA analysis was conducted to assess EV concentration, showing high EV recovery efficiency in both serum and urine, with particle counts ranging between 10^10^ and 10^11^ particles/mL (Figure 2B). Notably, in serum samples, EV concentration was significantly higher in the PCa group compared to the HC and BPH groups. In urine samples, while EV concentration was also elevated in the PCa group compared to HC, no statistically significant difference was observed when compared to BPH, likely due to substantial inter-individual variability within the BPH group.

qRT-PCR analysis revealed that miR−141 was significantly upregulated in PCa patients in the serum but not in the urine (Figure 2C,D). Additionally, miR−375, miR−21, and PCA3 lncRNA levels were elevated in patients compared to the HC in both serum and urine. Overall, except for PSA mRNA and T1-E4 mRNA, most candidate biomarkers showed diagnostic potential for PCa detection in both serum and urine through qRT-PCR validation.

To determine the concordance between qRT-PCR and TCLN-based RNA detection, serum-derived EVs were isolated from one HC and three PCa patients, followed by miR−141 quantification using both methods. After normalising to the HC sample, no significant differences in miR−141 levels were observed across the three PCa patients between the two methods, confirming strong consistency between qRT-PCR and TCLN methods (Figure 2E).

### Feasibility of TCLN-based EV RNA profiling for PCa diagnosis

**Figure 3.**
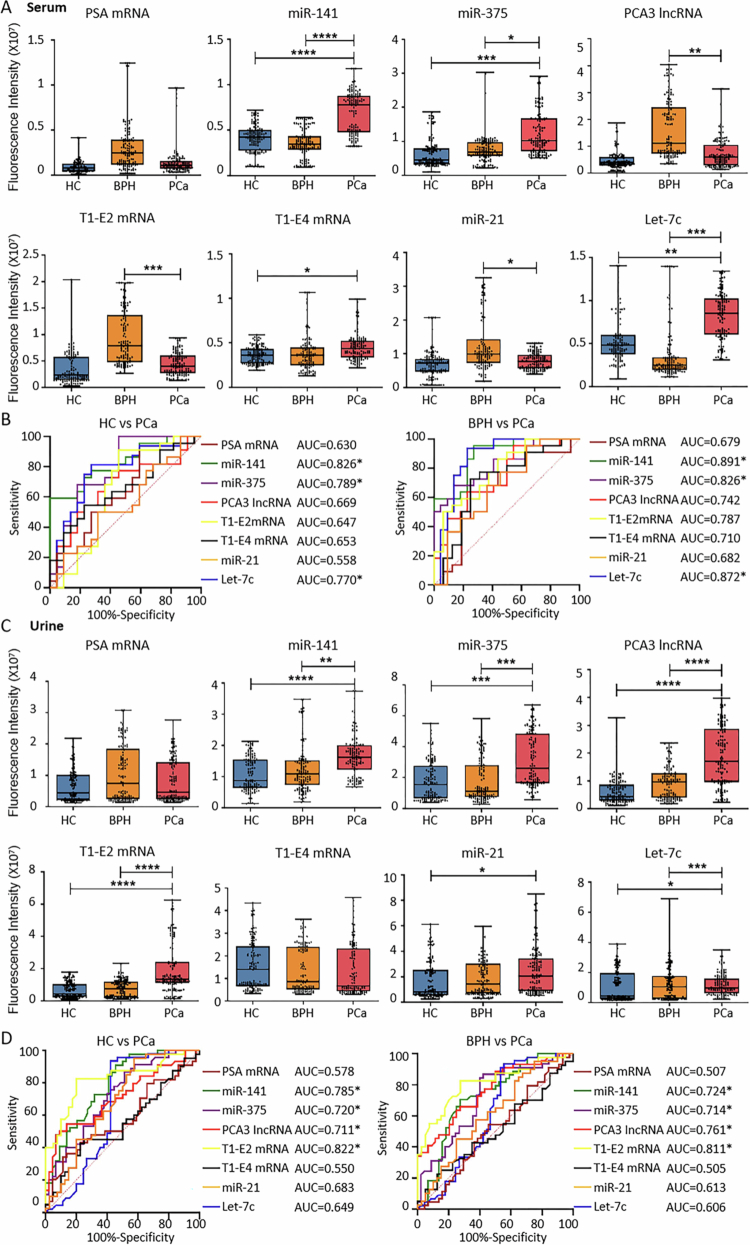
Expression of Candidate EV Biomarkers in Clinical Samples from the Training Cohort Detected by TCLN. (A) Expression levels of candidate EV biomarkers in serum samples from HC, BPH, and PCa patients, as measured by TCLN. Each group included 126 individuals. Student’s t-test was performed to compare differences between groups (*p <0.05, **p <0.01, **p <0.001). (B) ROC curves of the biomarkers presented in (A). Asterisks (*) denote biomarkers with higher expression in the PCa group than in HC or BPH, and with an AUC of ≥ 0.7. (C) Expression levels of candidate EV biomarkers in urine samples from HC, BPH, and PCa patients, as measured by TCLN. Each group included 135 individuals. Student’s t-test was performed to compare differences between groups (*p <0.05, **p <0.01, **p <0.001). (D) ROC curves of the biomarkers presented in (C). Asterisks (*) denote biomarkers with higher expression in the PCa group than in HC or BPH, and with an AUC of ≥ 0.7.

We examined the expression levels of the eight RNA biomarkers in the serum samples of participants in the training cohort using the TCLN technology (Figure 3A). The levels of miR−141, miR−375, and Let-7c, were significantly higher in PCa patients compared to HC and BPH patients. The PCA3 lncRNA, T1-E2 mRNA, and miR−21 levels of PCa patients were lower than BPH patients. The T1-E4 mRNA level in PCa patients was higher than the HCs. By plotting the receiver operating characteristic (ROC) curves, three serum RNA biomarkers showed area-under-the-curve (AUC) values of over 0.7 and were identified as potential indicators for early PCa diagnosis, including miR−141 (PCa vs. HC: AUC = 0.826; PCa vs. BPH: AUC = 0.891), miR−375 (PCa vs. HC: AUC = 0.789; PCa vs. BPH: AUC = 0.826), and Let-7c (PCa vs. HC: AUC = 0.770; PCa vs. BPH: AUC = 0.872) (Figure 3B).

In urine samples, the levels of miR−141, miR−375, PCA3 lncRNA, T1-E2 mRNA, and Let-7c, were significantly higher in PCa patients compared to HC and BPH patients (Figure 3C). Four RNA biomarkers in urine EVs had AUC values of over 0.7, including miR−141 (PCa vs. HC: AUC = 0.785; PCa vs. BPH: AUC = 0.724), miR−375 (PCa vs. HC: AUC = 0.720; PCa vs. BPH: AUC = 0.714), PCA3 lncRNA (PCa vs. HC: AUC = 0.711; PCa vs. BPH: AUC = 0.761), and T1-E2 mRNA (PCa vs. HC: AUC = 0.822; PCa vs. BPH: AUC = 0.811) (Figure 3D).

We further assessed the reproducibility and consistency of the TCLN assay. Specifically, all clinical samples were tested in triplicates across independent experimental runs. The coefficient of variation (CV) across these replicates ranged from 1.4% to 2.5%, with an average CV of 2.1% across both serum and urine samples, indicating high technical reproducibility. To evaluate batch-to-batch variability, all samples were randomly distributed and analysed across three independent experimental batches. No statistically significant differences in fluorescence intensities were observed between batches within each clinical group (Supplementary Table 1). These results are consistent with previously reported technical variation ranges for the TCLN platform (1–5% within chips and 5–20% across chips) and support the robustness of the assay.^37^^,^^38^

### Exploratory evaluation of EV RNA biomarkers using TCLN for predicting PCa staging and progression

**Figure 4.**
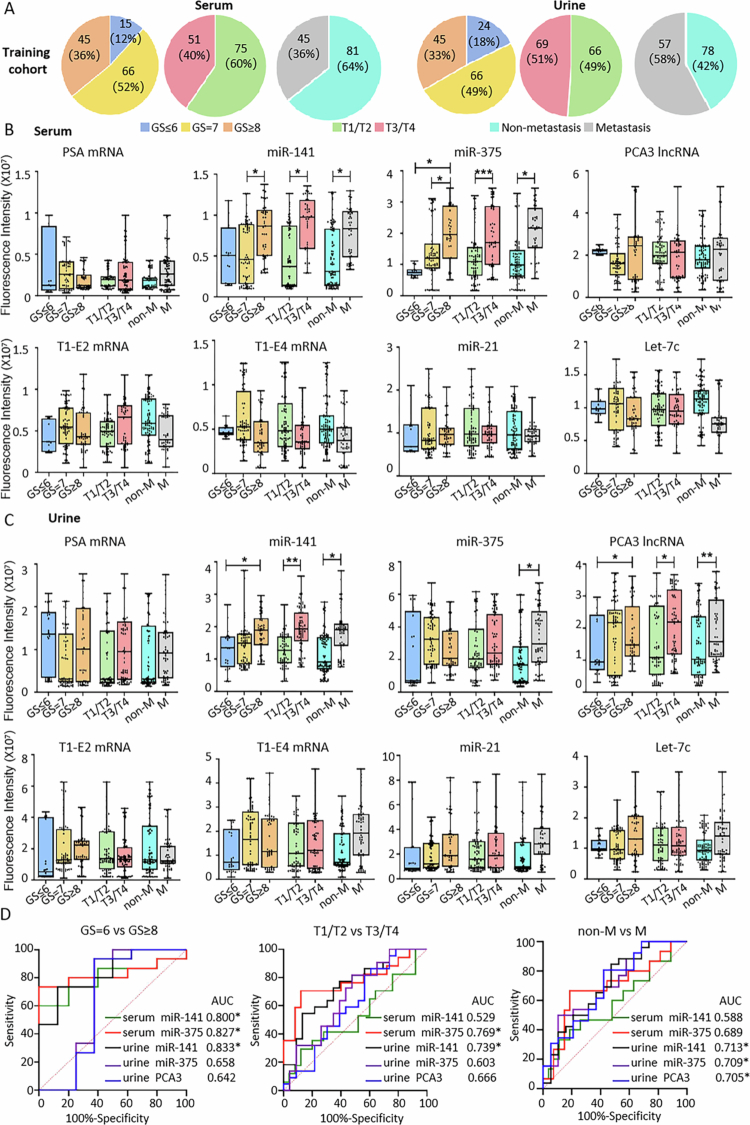
Expression of Candidate EV Biomarkers in Clinical Samples at Different Clinical Stages of PCa from the Training Cohort Detected by TCLN. (A) Gleason scores, tumour staging, and metastatic status of patients in the PCa group (126 serum samples and 135 urine samples). (B) and (C) Expression levels of EV biomarkers in the serum and urine of PCa patients at various clinical stages, as measured by TCLN. Boxplots display the interquartile range (IQR) of mean fluorescence intensity across different cohorts. Horizontal lines indicate the median values. Whiskers represent the maximum and minimum values. Student’s t-test was applied to assess differences between groups (*p <0.05, **p <0.01, **p <0.001). (D) ROC curves of the biomarkers presented in (B) and (C). Asterisks (*) indicate that the expression of a biomarker in the progression group (GS ≥ 8, T3/T4, metastasis) is higher than in the non-progression group (GS ≤ 7, T1/T2, non-metastasis), with AUC ≥ 0.7.

We then explored the potential of the EV RNA biomarkers in predicting PCa staging and progression. PCa patients in the training cohort were categorised by GS, tumour stage, and metastatic status derived from TNM staging at the time of sample collection (Figure 4A). In serum EV RNA biomarkers, miR−141 and miR−375 were elevated in PCa patients with GS ≥ 8, T3/T4, and metastases compared to those with GS ≤ 7, T1/T2, and no metastasis, respectively (Figure 4B). Among urine EV RNA biomarkers, miR−141 and PCA3 lncRNA were elevated in PCa patients with GS ≥ 8, T3/T4, and metastases compared to those with GS ≤ 6, T1/T2, and no metastasis, respectively (Figure 4C).

Serum miR−141, serum miR−375, and urine miR−141 showed AUC values of 0.800, 0.827, and 0.833 in differentiating PCa patients with GS = 6 and GS ≥ 8, respectively; serum miR−375 and urine miR−141 showed AUC values of 0.769 and 0.739 in differentiating patients with T1/T2 staging and those with T3/T4 staging, respectively; urine miR−141, miR−375, and PCA3 lncRNA achieved AUC values of 0.713, 0.709, and 0.705 in differentiating metastatic and non-metastatic patients, respectively (Figure 4D). The optimal cut-off values, sensitivity, specificity, and Youden Index values of these RNA markers are summarised in Table 3.

**Table 3.** The biomarkers selected in the training cohort and their cut-off values determined by the maximum YI.

Clinical application	Sample	RNA marker	MFI Cut-off (X10^7)	Sensitivity (%)	Specificity (%)	Youden Index
PCa early diagnosis (PCa vs HC&BPH)	serum	miR−141	0.67	83.87	85.81	0.70
		miR−375	0.82	82.58	77.97	0.61
		let-7c	0.69	89.52	82.9	0.72
	urine	miR−141	1.41	86.29	82.26	0.69
		miR−375	1.65	85.48	80.65	0.66
		PCA3 lncRNA	1.32	87.9	79.03	0.67
		T1-E2 mRNA	1.14	83.06	75.81	0.59
PCa progression diagnosis (GS ≤ 6 vs GS ≥ 8)	serum	miR−141	0.74	84.84	79.65	0.64
		miR−375	1.12	82.81	89.68	0.72
	urine	miR−141	1.43	85.81	79.68	0.65
		miR−375	1.56	88.06	77.42	0.65
		PCA3 lncRNA	1.58	79.42	83.06	0.62
PCa progression diagnosis (T1/T2 vs T3/T4)	serum	miR−141	0.76	82.45	78.23	0.61
		miR−375	1.54	85.81	88.87	0.75
	urine	miR−141	1.76	85.81	77.82	0.64
		miR−375	1.45	87.42	77.26	0.65
		PCA3 lncRNA	1.68	88.39	75.65	0.64

### Performance of EV RNA biomarkers in the validation cohort

**Figure 5.**
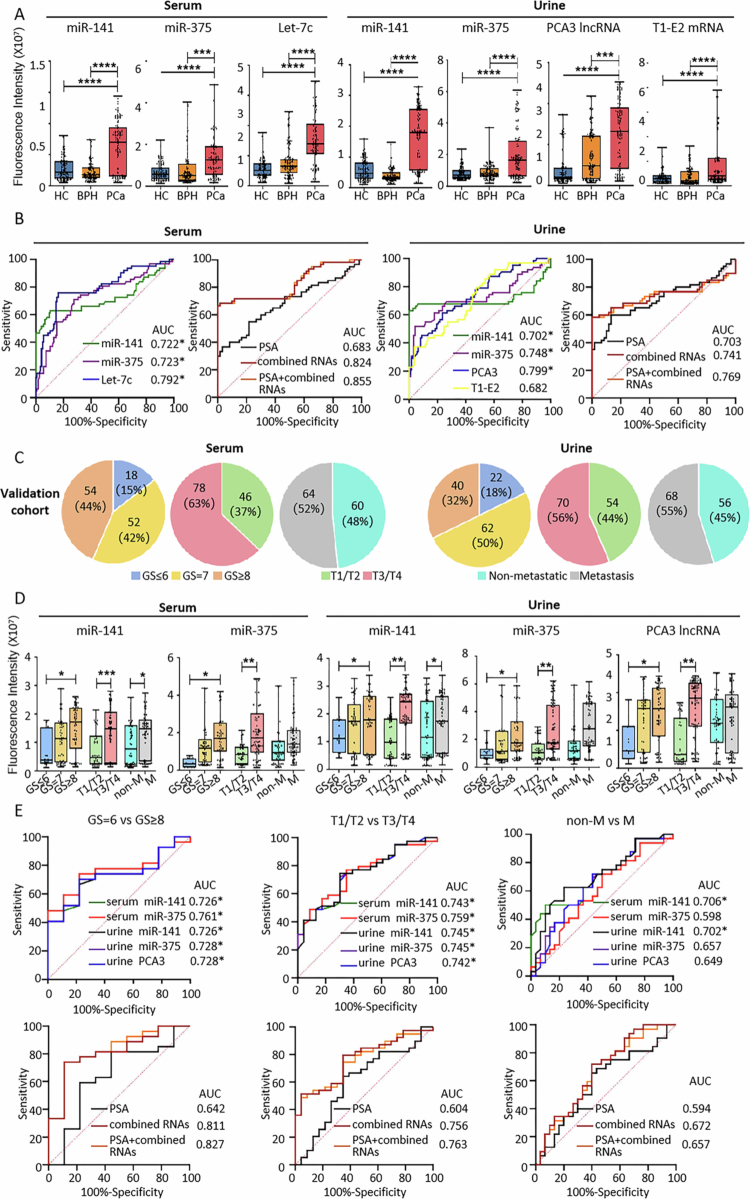
Expression Levels and Diagnostic Performance of EV Biomarkers in the Validation Cohort Clinical Samples. (A) Expression levels of the selected serum and urine EV biomarkers in the validation cohort. (B) ROC curves of individual EV RNA biomarkers, combined EV RNA biomarkers, and PSA in PCa diagnosis. (C) Gleason scores, tumour staging, and metastatic status of patients in the PCa group (124 serum samples and 124 urine samples). (D) Expression levels of the selected serum and urine EV biomarkers in the validation cohort, grouped by Gleason scores, tumour staging, and metastatic status. (E) ROC curves of individual EV RNA biomarkers, combined EV RNA biomarkers, and PSA in PCa staging and progression prediction.

To evaluate the reproducibility of the identified EV RNA biomarkers in an independent sample set, TCLN detection was conducted in a separate validation cohort comprising 124 cases per group for both serum and urine. The diagnostic values of serum miR−141, miR−375, Let-7c, and urine miR−141, miR−375, PCA3 lncRNA, and T1-E2 mRNA remained significant in the validation cohort (Figure 5A). We further combined the RNA biomarkers and examined its performance in diagnosis compared with PSA. Within the validation cohort, the combined serum RNAs showed a diagnostic AUC of 0.824, which was higher than any of the RNAs alone (miR−141, miR−375, and Let-7c), and PSA (AUC = 0.683) (Figure 5B). Further combining the serum RNAs with PSA resulted in a slight increase in AUC (AUC = 0.855). Combining the four urine RNAs resulted in an overall AUC of 0.741, which was still higher than PSA (AUC = 0.703). Further combining these urine RNAs with PSA achieved an AUC of 0.769, but this was still lower than PCA3 lncRNA alone (AUC = 0.799) (Figure 5B). To ensure the reliability of the above models, we also conducted a post-hoc power analysis based on Cohen’s d effect size. At a significance level of *α* = 0.05 and an effect size of d = 0.6, the statistical power (1 − *β*) reached 91.2% and 92.5% for the serum and urine models, respectively, indicating sufficient sample size for robust inference.

The staging and progression categories of the validation cohort samples are shown in Figure 5C. In staging and progression prediction, all five RNA biomarkers (serum miR−141, serum miR−375, urine miR−141, urine miR−375, and urine PCA3 lncRNA) maintained their trends and achieved AUC values of over 0.7 in differentiating patients with GS = 6 and GS ≥ 8, as well as in differentiating those with T1/T2 and T3/T4 staging (Figure 5D,E). Only serum miR−141 and urine miR−141 achieved AUC values of over 0.7 in differentiating metastatic and non-metastatic patients. Combining these RNA biomarkers significantly improved the AUCs in differentiating the three patient groups, with AUCs being 0.811, 0.756, and 0.672, respectively, all of which surpassed the performance of PSA alone. Further combining them with PSA resulted in slight increases in AUC in differentiating GS = 6 vs GS ≥ 8 (AUC = 0.827) and T1/T2 vs T3/T4 groups (AUC = 0.763), but not in metastasis vs non-metastasis groups (AUC = 0.657).

## Discussion

In this study, we utilised the TCLN biochip-based approach to detect EV RNA biomarkers for the early diagnosis and progression prediction of PCa. We screened eight RNA targets, including miRNAs, lncRNAs, and mRNA fusion transcripts, in both serum- and urine-derived EVs from PCa patients, BPH patients, and healthy controls. We first validated the presence of RNA-carrying EVs in prostate cancer cell lines using the TCLN technology. We then confirmed the consistency between qRT-PCR and TCLN results within a small cohort of PCa patients, BPH patients and controls, and identified several upregulated markers, including miR−141, miR−375, and PCA3 lncRNA, in the serum and urine samples of PCa patients. In particular, TCLN results showed that serum miR−141, miR−375, and Let-7c, as well as urine miR−141, miR−375, PCA3 lncRNA, and T1-E2 mRNA, achieved AUC values above 0.7 in differentiating PCa patients from BPH patients and healthy controls, demonstrating their strong diagnostic potential. Furthermore, EV RNA biomarkers showed promising predictive values in PCa staging and progression: serum miR−141 and miR−375, along with urine miR−141 and PCA3 lncRNA, correlated with higher GS, advanced tumour stages, and metastatic status. These findings were consistently reproduced in an independent validation cohort. In addition, combining EV RNA biomarkers resulted in improved AUC values in both PCa diagnosis and staging and progression prediction, surpassing those of PSA alone, suggesting that these EV RNAs may serve as promising biomarkers for early detection and risk stratification of PCa.

Compared to the conventional qRT-PCR approach, the TCLN platform offers several technical advantages for EV RNA detection.^37^^,^^40^ qRT-PCR relies on the amplification of longer RNA segments (typically over 100 nucleotides) and requires two intact primer binding sites, which can pose challenges when working with fragmented or low-abundance transcripts commonly found in EVs. In contrast, TCLN utilises fluorescent MBs that hybridise directly with short RNA sequences (20–30 nucleotides), enabling the detection of both full-length and degraded RNA fragments without amplification. This feature enhances its compatibility with the diverse and heterogeneous RNA species encapsulated in EVs. Additionally, TCLN requires significantly less sample input and eliminates the need for labour-intensive RNA extraction and reverse transcription steps.^40^ To further improve clinical feasibility, we employed precipitation-based EV isolation instead of ultracentrifugation. Our results showed that EVs isolated by these clinically practical methods were compatible with TCLN detection. Moreover, colocalization experiments using prostate cancer cell-derived EVs confirmed that MB-targeted RNAs such as miR−141 co-localised with the exosomal marker CD63, supporting the biological relevance and detection specificity of the TCLN platform. Importantly, TCLN is a single-EV resolution platform that allows for co-localisation analyses of multiple RNA and protein markers on the same vesicle.^37^ In contrast, qRT-PCR is a bulk analysis method that averages signals across the entire EV population, potentially masking biologically relevant subpopulations. By preserving the spatial and molecular heterogeneity of EVs, TCLN may enable a more nuanced profiling of disease-related EV subtypes, which may prove valuable in identifying rare but clinically significant biomarker signals. Given the well-established associations between EV heterogeneity and cancer presence and aggressiveness,^55^ we believe that single-EV-level TCLN imaging holds strong potential to yield critical clinical insights. Although this capability was not fully utilised in the present proof-of-concept study, we plan to leverage single-EV data in future investigations to perform heterogeneity-oriented analyses incorporating EV size, fluorescence intensity, and co-localisation patterns, and to apply distribution-specific or clustering algorithms to characterise distinct EV subtypes. For example, correlating co-localised EV markers, specific RNA signals, clinical characteristics, and disease stages may provide some mechanistic insights into the origin of EVs with certain profiles. In particular, we also aim to investigate rare EV populations that may carry strong diagnostic or prognostic value to provide more clinical insights. Such analyses would fully realise the advantages of the TCLN platform over conventional bulk measurements, which average out molecular heterogeneity and mask clinically relevant subpopulation signals.

In this study, we demonstrated the clinical utility of EV RNA biomarkers detected by TCLN for both early PCa diagnosis and disease progression assessment. Notably, several of the top-performing markers, such as miR−141, miR−375, and PCA3, are well-documented in prior studies as PCa-associated RNAs (Supplementary Table 2),^16^^,^^26^^,^^45^ reinforcing the validity of our results. Beyond diagnosis, we extended our research to investigate the risk stratification values of EV RNAs, demonstrating their correlations with GS, tumour staging, and metastatic status. Nevertheless, while most of our results align with previous reports, certain inconsistencies were observed. For instance, the utility of Let-7c in PCa remains controversial; although upregulated in our cohort, other studies have reported tumour-suppressive roles for this RNA, which is typically downregulated in PCa.^48^^,^^56^ Further investigations may be required to understand the abnormal expression pattern of Let-7c in our cohort. Additionally, detection performance for metastasis was more modest, and distinguishing GS = 7 from higher-grade PCa remained a challenge, which was likely due to biological overlap and the relatively small number of GS ≤ 6 cases in our sample cohorts.

This study presents several strengths. Firstly, we simultaneously assessed EV RNA biomarkers in both serum and urine, providing complementary insights into PCa biology and expanding the potential for non-invasive diagnostics. Secondly, the use of the TCLN platform enabled multiplex, amplification-free detection of small RNAs at the single-EV level, facilitating more efficient and scalable workflows compared to qRT-PCR. Thirdly, the integration of diagnostic and risk stratification analyses in both discovery and validation cohorts reinforces the clinical relevance and reproducibility of our findings.

Several limitations should also be acknowledged. The sample size, while moderate, may still limit statistical power, particularly in subgroup comparisons such as GS ≤ 6 versus GS = 7 or metastatic versus localised disease. In addition, although the TCLN platform allows for the detection of fragmented RNAs, it currently does not provide absolute quantification or direct insights into RNA function. We also observed variability in urine-derived EV purity, which may have impacted signal consistency and contributed to lower diagnostic performance compared to serum-based models. Moreover, while our panel includes both miRNA and mRNA/lncRNA targets to enhance biological coverage, we observed partial inconsistencies between TCLN and qRT-PCR results for certain biomarkers. This may be attributed to the surface chemistry of the TCLN platform, which could selectively capture subpopulations of EVs, thereby introducing detection bias relative to bulk RNA extraction methods. Furthermore, while the two-tier detection strategy of TCLN provides target sequence specificity, additional validation studies are needed to fully characterise the specificity of EV capture and confirm exclusion of non-EV particles. From a technical perspective, while we only validated EV enrichment using CD63 immunofluorescence due to instrument channel limitations, incorporating additional markers such as CD9 and CD81 in future work would improve the characterisation of the overall EV population.^57^ Moreover, careful comparison and optimisation of EV isolation methods across different sample types should also be performed to maximise assay performance and reduce the impact of sample-derived contaminants, particularly in urine-based EV analysis. The overall SNR across clinical samples remains to be systematically characterised and optimised to improve the robustness and reproducibility of the pipeline. Notably, the current EV RNA panel showed limited ability to differentiate Gleason score 7 cases from other PCa grades, reflecting both the biological heterogeneity of intermediate-risk disease and the limitations of the marker selection in this study. Incorporating analyses based on EV heterogeneity may help uncover potential associations between EV subpopulation dynamics and Gleason score, thereby improving the prognostic value of single-EV profiling in prostate cancer. In addition, validation of the full EV RNA panel with alternative methods such as qRT-PCR was only performed for miR−141 as a representative marker in this study, and future work should include systematic cross-platform validation for the entire biomarker panel. Future studies should also focus on expanding cohort size, especially across diverse clinical subgroups, and evaluating longitudinal EV RNA dynamics during disease progression and treatment. In addition, a comprehensive and systematic large-scale evaluation of candidate EV RNA biomarkers could further refine the marker panel, support biomarker discovery, and reduce potential selection bias: the optimal biomarker configuration, whether based on single markers or combined panels, should ultimately be established in larger, multi-centre validation studies to ensure clinical robustness. Efforts to integrate TCLN-based EV profiling with other modalities, such as proteomics, genomics, or imaging, may further enhance its diagnostic resolution and translational value in precision oncology.

In light of the current singleplex limitations, future technical improvements to the TCLN platform may greatly enhance its clinical utility. One promising direction involves designing molecular beacons with distinct fluorescent labels and leveraging the multichannel scanning capabilities of TIRFM. This multiplexed strategy would enable simultaneous detection of multiple RNA targets in the same well, thereby increasing throughput, reducing sample consumption, and facilitating rapid profiling of complex EV RNA signatures in clinical applications. Such advancements are expected to expand the diagnostic resolution and translational value of TCLN in precision oncology.

## Conclusion

In summary, we utilised a TCLN biochip-based method for the detection of EV RNA biomarkers in serum and urine samples from PCa patients. Our results demonstrate that specific EV RNAs, such as miR−141, miR−375, and PCA3 lncRNA, are effective in distinguishing PCa from non-cancer controls and in stratifying patients by tumour grade and stage. These findings highlight the potential of TCLN-enabled EV RNA profiling as a powerful tool for early diagnosis and risk assessment in PCa, particularly using biomarkers such as miR−141, miR−375, and Let-7c. Further large-scale, longitudinal studies are required to validate the clinical values of these biomarkers.

## Acknowledgments

The authors would like to thank editors and reviewers for their insightful suggestions and careful reading of manuscript.

## Supplementary Material

Supplementary Material Supplementary Tables_revised.docx

## Data Availability

The data that support the findings of this study are not publicly available due to the presence of sensitive and/or confidential information, including patient-related data. Access to these data may be granted upon reasonable request and subject to appropriate ethical and institutional approvals. Researchers interested in accessing the data should contact the author at dyj2202010066@163.com.

## References

[1] Rawla P. Epidemiology of prostate cancer. World J Oncol. 2019;10:63–89. doi: 10.14740/wjon1191. 31068988 PMC6497009

[2] Litwin MS, Tan HJ. The diagnosis and treatment of prostate cancer: a review. Jama. 2017;317:2532–2542. doi: 10.1001/jama.2017.7248. 28655021

[3] Sung H, Ferlay J, Siegel RL, Laversanne M, Soerjomataram I, Jemal A, Bray F. Global cancer statistics 2020: GLOBOCAN estimates of incidence and mortality worldwide for 36 cancers in 185 countries. CA Cancer J Clin. 2021;71:209–249. doi: 10.3322/caac.21660. 33538338

[4] Carlsson SV, Vickers AJ. Screening for prostate cancer. Med Clin North Am. 2020;104:1051–1062. doi: 10.1016/j.mcna.2020.08.007. 33099450 PMC8287565

[5] Harding TA, Martin RM, Merriel SW, Jones R, O'Sullivan JM, Kirby M, O’Sullivan JM, Olajide O, Norman A, Bhatt J, et al. Optimising the use of the prostate- specific antigen blood test in asymptomatic men for early prostate cancer detection in primary care: report from a UK clinical consensus. Br J Gen Pract. 2024;74:e534–e543. doi: 10.3399/BJGP.2023.0586. 39038964 PMC11289937

[6] Carlsson SV, Roobol MJ. Improving the evaluation and diagnosis of clinically significant prostate cancer in 2017. Curr Opin Urol. 2017;27:198–204. doi: 10.1097/MOU.0000000000000382. 28221219 PMC5381721

[7] Sheta M, Taha EA, Lu Y, Eguchi T. Extracellular vesicles: new classification and tumor immunosuppression. Biology (Basel). 2023;12:110. doi: 10.3390/biology12010110. 36671802 PMC9856004

[8] Doyle LM, Wang MZ. Overview of extracellular vesicles, their origin, composition, purpose, and methods for exosome isolation and analysis. Cells. 2019;8:727. doi: 10.3390/cells8070727. 31311206 PMC6678302

[9] Kim KM, Abdelmohsen K, Mustapic M, Kapogiannis D, Gorospe M. RNA in extracellular vesicles. Wiley Interdiscip Rev RNA. 2017;8, 10.1002/wrna.1413. PMC5474163 28130830

[10] Pink RC, Beaman EM, Samuel P, Brooks SA, Carter DRF. Utilising extracellular vesicles for early cancer diagnostics: benefits, challenges and recommendations for the future. Br J Cancer. 2022;126:323–330. doi: 10.1038/s41416-021-01668-4. 35013578 PMC8810954

[11] Yu L, Sui B, Fan W, Lei L, Zhou L, Yang L, Diao Y, Zhang Y, Li Z, Liu J, et al. Exosomes derived from osteogenic tumor activate osteoclast differentiation and concurrently inhibit osteogenesis by transferring COL1A1-targeting miRNA-92a-1-5p. J Extracell Vesicles. 2021;10:e12056. doi: 10.1002/jev2.12056. 33489015 PMC7812369

[12] Bryant RJ, Pawlowski T, Catto JW, Marsden G, Vessella RL, Rhees B, Kuslich C, Visakorpi T, Hamdy FC. Changes in circulating microRNA levels associated with prostate cancer. Br J Cancer. 2012;106:768–774. doi: 10.1038/bjc.2011.595. 22240788 PMC3322952

[13] Hendriks RJ, Dijkstra S, Jannink SA, Steffens MG, van Oort IM, Mulders PF, Schalken JA. Comparative analysis of prostate cancer specific biomarkers PCA3 and ERG in whole urine, urinary sediments and exosomes. Clin Chem Lab Med. 2016;54:483–492. doi: 10.1515/cclm-2015-0599. 26630694

[14] Pellegrini KL, Patil D, Douglas KJS, Lee G, Wehrmeyer K, Torlak M, Clark J, Cooper CS, Moreno CS, Sanda MG. Detection of prostate cancer-specific transcripts in extracellular vesicles isolated from post-DRE urine. Prostate. 2017;77(9):990–999. doi: 10.1002/pros.23355. 28419548 PMC5907935

[15] Foj L, Ferrer F, Serra M, Arévalo A, Gavagnach M, Giménez N, Filella X. Exosomal and non-exosomal urinary mirnas in prostate cancer detection and prognosis. Prostate. 2017;77:573–583. doi: 10.1002/pros.23295. 27990656

[16] Li Z, Ma YY, Wang J, Zeng XF, Li R, Kang W, Hao XK. Exosomal microRNA-141 is upregulated in the serum of prostate cancer patients. Onco Targets Ther. 2016;9:139–148. 26770063 10.2147/OTT.S95565 PMC4706124

[17] Huang X, Yuan T, Liang M, Du M, Xia S, Dittmar R, Wang D, See W, Costello BA, Quevedo F, et al. Exosomal miR-1290 and miR-375 as prognostic markers in castration-resistant prostate cancer. Eur Urol. 2015;67:33–41. doi: 10.1016/j.eururo.2014.07.035. 25129854 PMC4252606

[18] Samsonov R, Shtam T, Burdakov V, Glotov A, Tsyrlina E, Berstein L, Nosov A, Evtushenko V, Filatov M, Malek A. Lectin-induced agglutination method of urinary exosomes isolation followed by mi-RNA analysis: application for prostate cancer diagnostic. Prostate. 2016;76:68–79. doi: 10.1002/pros.23101. 26417675

[19] Donovan MJ, Noerholm M, Bentink S, Belzer S, Skog J, O'Neill V, Cochran JS, Brown GA. A molecular signature of PCA3 and ERG exosomal RNA from non-DRE urine is predictive of initial prostate biopsy result. Prostate Cancer Prostatic Dis. 2015;18:370–375. doi: 10.1038/pcan.2015.40. 26345389

[20] Koppers-Lalic D, Hackenberg M, de Menezes R, Misovic B, Wachalska M, Geldof A, Zini N, de Reijke T, Wurdinger T, Vis A, et al. Non‑invasive prostate cancer detection by measuring miRNA variants (isomiRs) in urine extracellular vesicles. Oncotarget. 2016;7:22566–22578. doi: 10.18632/oncotarget.8124. 26992225 PMC5008382

[21] McKiernan J, Donovan MJ, O'Neill V, Bentink S, Noerholm M, Belzer S, O’Neill V, Skog J, Kattan MW, Partin A, et al. A novel urine exosome gene expression assay to predict high-grade prostate cancer at initial biopsy. JAMA Oncol. 2016;2:882–889. doi: 10.1001/jamaoncol.2016.0097. 27032035

[22] Motamedinia P, Scott AN, Bate KL, Sadeghi N, Salazar G, Shapiro E, Ahn J, Lipsky M, Lin J, Hruby GW, et al. Urine exosomes for non-invasive assessment of gene expression and mutations of prostate cancer. PLoS One. 2016;11:e0154507. doi: 10.1371/journal.pone.0154507. 27144529 PMC4856378

[23] Smith SF, Brewer DS, Hurst R, Cooper CS. Applications of urinary extracellular vesicles in the diagnosis and active surveillance of prostate cancer. Cancers (Basel). 2024;16:1717. doi: 10.3390/cancers16091717. 38730670 PMC11083542

[24] Kanavarioti A, Rehman MH, Qureshi S, Rafiq A, Sultan M. High sensitivity and specificity platform to validate microrna biomarkers in cancer and human diseases. Noncoding RNA. 2024;10:42. doi: 10.3390/ncrna10040042. 39051376 PMC11270241

[25] Duca RB, Massillo C, Dalton GN, Farré PL, Graña KD, Gardner K, De Siervi A. MiR-19b-3p and miR-101-3p as potential biomarkers for prostate cancer diagnosis and prognosis. Am J Cancer Res. 2021;11:2802–2820. 34249429 PMC8263646

[26] Lemos AEG, Matos ADR, Ferreira LB, Gimba ERP. The long non-coding RNA PCA3: an update of its functions and clinical applications as a biomarker in prostate cancer. Oncotarget. 2019;10:6589–6603. doi: 10.18632/oncotarget.27284. 31762940 PMC6859920

[27] Qin X, Zhu W, Lu A, Wang G, Ye X, Weng G. Long non-coding RNA SAP30L-AS1 promotes prostate cancer growth through repressing SAP30L. Gene. 2019;690:120–128. doi: 10.1016/j.gene.2018.12.047. 30599235

[28] Kidd SG, Carm KT, Bogaard M, Olsen LG, Bakken AC, Løvf M, Lothe RA, Axcrona K, Skotheim RI. High expression of SCHLAP1 in primary prostate cancer is an independent predictor of biochemical recurrence, despite substantial heterogeneity. Neoplasia. 2021;23:634–641. doi: 10.1016/j.neo.2021.05.012. 34107378 PMC8192444

[29] Bajo-Santos C, Brokāne A, Zayakin P, Endzeliņš E, Soboļevska K, Belovs A, Jansons J, Sperga M, Llorente A, Radoviča-Spalviņa I, et al. Plasma and urinary extracellular vesicles as a source of RNA biomarkers for prostate cancer in liquid biopsies. Front Mol Biosci. 2023;10:980433. doi: 10.3389/fmolb.2023.980433. 36818049 PMC9935579

[30] Choi JW, Kim SC, Hong SH, Lee HJ. Secretable small RNAs via outer membrane vesicles in periodontal pathogens. J Dent Res. 2017;96:458–466. doi: 10.1177/0022034516685071. 28068479

[31] Kim HJ, Rames MJ, Goncalves F, Kirschbaum CW, Roskams-Hieter B, Spiliotopoulos E, Briand J, Doe A, Estabrook J, Wagner JT, et al. Selective enrichment of plasma cell-free messenger RNA in cancer-associated extracellular vesicles. Commun Biol. 2023;6:885. doi: 10.1038/s42003-023-05232-z. 37644220 PMC10465482

[32] Koeppen K, Hampton TH, Jarek M, Scharfe M, Gerber SA, Mielcarz DW, Demers EG, Dolben EL, Hammond JH, Hogan DA, et al. A novel mechanism of host-pathogen interaction through sRNA in bacterial outer membrane vesicles. PLoS Pathog. 2016;12:e1005672. doi: 10.1371/journal.ppat.1005672. 27295279 PMC4905634

[33] Malabirade A, Habier J, Heintz-Buschart A, May P, Godet J, Halder R, Etheridge A, Galas D, Wilmes P, Fritz JV. The RNA complement of outer membrane vesicles from salmonella enterica serovar typhimurium under distinct culture conditions. Front Microbiol. 2018;9:2015. doi: 10.3389/fmicb.2018.02015. 30214435 PMC6125333

[34] Dellar ER, Hill C, Melling GE, Carter DRF, Baena-Lopez LA. Unpacking extracellular vesicles: RNA cargo loading and function. J Extracell Biol. 2022;1:e40. doi: 10.1002/jex2.40. 38939528 PMC11080855

[35] De Sousa KP, Rossi I, Abdullahi M, Ramirez MI, Stratton D, Inal JM. Isolation and characterization of extracellular vesicles and future directions in diagnosis and therapy. Wiley Interdiscip Rev Nanomed Nanobiotechnol. 2023;15:e1835. doi: 10.1002/wnan.1835. 35898167 PMC10078256

[36] Miceli RT, Chen TY, Nose Y, Tichkule S, Brown B, Fullard JF, Saulsbury MD, Heyliger SO, Gnjatic S, Kyprianou N, et al. Extracellular vesicles, RNA sequencing, and bioinformatic analyses: challenges, solutions, and recommendations. J Extracell Vesicles. 2024;13:e70005. doi: 10.1002/jev2.70005. 39625409 PMC11613500

[37] Wu Y, Kwak KJ, Agarwal K, Marras A, Wang C, Mao Y, Huang X, Ma J, Yu B, Lee R, et al. Detection of extracellular RNAs in cancer and viral infection via tethered cationic lipoplex nanoparticles containing molecular beacons. Anal Chem. 2013;85:11265–11274. doi: 10.1021/ac401983w. 24102152 PMC4121114

[38] Lee LJ, Yang Z, Rahman M, Ma J, Kwak KJ, McElroy J, Shilo K, Goparaju C, Yu L, Rom W, et al. Extracellular mRNA detected by tethered lipoplex nanoparticle biochip for lung Adenocarcinoma detection. Am J Respir Crit Care Med. 2016;193:1431–1433. doi: 10.1164/rccm.201511-2129LE. 27304243 PMC4910892

[39] Zhou J, Qu G, Zhang G, Wu Z, Liu J, Yang D, Li J, Chang M, Zeng H, Hu J, et al. Glycerol kinase 5 confers gefitinib resistance through SREBP1/SCD1 signaling pathway. J Exp Clin Cancer Res. 2019;38:96. doi: 10.1186/s13046-019-1057-7. 30791926 PMC6385389

[40] Liu C, Kannisto E, Yu G, Yang Y, Reid ME, Patnaik SK, Wu Y. Non-invasive Detection of exosomal MicroRNAs via tethered cationic lipoplex nanoparticles (tCLN) biochip for lung cancer early detection. Front Genet. 2020;11:258. doi: 10.3389/fgene.2020.00258. 32265989 PMC7100709

[41] Zhou J, Kwak KJ, Wu Z, Yang D, Li J, Chang M, Song Y, Zeng H, Lee LJ, Hu J, et al. PLAUR confers resistance to Gefitinib through EGFR/P-AKT/Survivin signaling pathway. Cell Physiol Biochem. 2018;47:1909–1924. doi: 10.1159/000491071. 29961070

[42] Harding MA, Yavuz H, Gathmann A, Upson S, Swiatecka-Urban A, Erdbrügger U. Uromodulin and the study of urinary extracellular vesicles. J Extracell Biol. 2024;3:e70022. doi: 10.1002/jex2.70022. 39582686 PMC11583080

[43] Selth LA, Townley SL, Bert AG, Stricker PD, Sutherland PD, Horvath LG, Goodall GJ, Butler LM, Tilley WD. Circulating microRNAs predict biochemical recurrence in prostate cancer patients. Br J Cancer. 2013;109:641–650. doi: 10.1038/bjc.2013.369. 23846169 PMC3738112

[44] Singh VK, Rajak N, Singh Y, Singh AK, Giri R, Garg N. Role of MicroRNA-21 in prostate cancer progression and metastasis: molecular mechanisms to therapeutic targets. Ann Surg Oncol. 2024;31:4795–4808. doi: 10.1245/s10434-024-15453-z. 38758485

[45] Joković SM, Dobrijević Z, Kotarac N, Filipović L, Popović M, Korać A, Vuković I, Savić-Pavićević D, Brajušković G. MiR-375 and miR-21 as potential biomarkers of prostate cancer: comparison of matching samples of plasma and exosomes. Genes (Basel). 2022;13:2320. doi: 10.3390/genes13122320. 36553586 PMC9778022

[46] Jain G, Das P, Ranjan P, Neha, Valderrama F, Cieza-Borrella C. Urinary extracellular vesicles miRNA-A new era of prostate cancer biomarkers. Front Genet. 2023;14:1065757. doi: 10.3389/fgene.2023.1065757. 36741322 PMC9895092

[47] Hatano K, Fujita K. Extracellular vesicles in prostate cancer: a narrative review. Transl Androl Urol. 2021;10:1890–1907. doi: 10.21037/tau-20-1210. 33968677 PMC8100827

[48] Mulholland EJ, Green WP, Buckley NE, McCarthy HO. Exploring the potential of MicroRNA Let-7c as a therapeutic for prostate cancer. Mol Ther Nucleic Acids. 2019;18:927–937. doi: 10.1016/j.omtn.2019.09.031. 31760377 PMC6883330

[49] Prensner JR, Zhao S, Erho N, Schipper M, Iyer MK, Dhanasekaran SM, Magi-Galluzzi C, Mehra R, Sahu A, Siddiqui J, et al. RNA biomarkers associated with metastatic progression in prostate cancer: a multi-institutional high-throughput analysis of SChLAP1. Lancet Oncol. 2014;15:1469–1480. doi: 10.1016/S1470-2045(14)71113-1. 25456366 PMC4559342

[50] Tomlins SA, Rhodes DR, Perner S, Dhanasekaran SM, Mehra R, Sun XW, Varambally S, Cao X, Tchinda J, Kuefer R, et al. Recurrent fusion of TMPRSS2 and ETS transcription factor genes in prostate cancer. Science. 2005;310:644–648. doi: 10.1126/science.1117679. 16254181

[51] Clark J, Merson S, Jhavar S, Flohr P, Edwards S, Foster CS, Eeles R, Martin FL, Phillips DH, Crundwell M, et al. Diversity of TMPRSS2-ERG fusion transcripts in the human prostate. Oncogene. 2007;26:2667–2673. doi: 10.1038/sj.onc.1210070. 17043636

[52] Fluss R, Faraggi D, Reiser B. Estimation of the Youden Index and its associated cutoff point. Biom J. 2005;47:458–472. doi: 10.1002/bimj.200410135. 16161804

[53] Diao Y, Zhu B, Ding T, Li R, Li J, Yang L, Zhou L, Hao X, Liu J. Tumor-derived extracellular vesicle nucleic acids as promising diagnostic biomarkers for prostate cancer. Front Oncol. 2023;13:1201554. doi: 10.3389/fonc.2023.1201554. 37456240 PMC10338955

[54] Okada-Tsuchioka M, Kajitani N, Omori W, Kurashige T, Boku S, Takebayashi M. Tetraspanin heterogeneity of small extracellular vesicles in human biofluids and brain tissue. Biochem Biophys Res Commun. 2022;627:146–151. doi: 10.1016/j.bbrc.2022.08.025. 36037746

[55] Silva TF, Hutchins E, Zhao W, Ciani Y, Kim M, Ko E, Mariscal J, Qiu Z, Bedier F, Kittel A, et al. Extracellular vesicle heterogeneity through the lens of multiomics. Cell Reports Medicine. 2025;6:102161. doi: 10.1016/j.xcrm.2025.102161. 40482644 PMC12281359

[56] Nadiminty N, Tummala R, Lou W, Zhu Y, Zhang J, Chen X, deVere White RW, Kung H, Evans CP, Gao AC. MicroRNA let-7c suppresses androgen receptor expression and activity via regulation of Myc expression in prostate cancer cells. J Biol Chem. 2012;287:1527–1537. doi: 10.1074/jbc.M111.278705. 22128178 PMC3256915

[57] Jennrich S, Pelzer M, Tertel T, Koska B, Vüllings M, Thakur BK, Jendrossek V, Timmermann B, Giebel B, Rudner J. CD9- and CD81-positive extracellular vesicles provide a marker to monitor glioblastoma cell response to photon-based and proton-based radiotherapy. Front Oncol. 2022;12:947439. doi: 10.3389/fonc.2022.947439. 36203458 PMC9530604

