# Supplementary material for: A novel biochip-based liquid biopsy for extracellular vesicle RNA detection in prostate cancer
Source: Cancer Biol Ther. 2025 Dec 3;26(1):2593744. doi: 10.1080/15384047.2025.2593744 (PMC12688251; doi:10.1080/15384047.2025.2593744)
Supplement: Supplementary Material — Supplementary Tables_revised.docx [file KCBT_A_2593744_SM9506.docx]

**Supplementary Table 1.** Kruskal-Wallis test p-values between different test batches. HC, healthy control. BPH, benign prostatic hyperplasia. PCa, prostate cancer.

| Sample type | Group | Kruskal-Wallis test p-value |
| --- | --- | --- |
| Serum | All | 0.398 |
|  | HC | 0.412 |
|  | BPH | 0.389 |
|  | PCa | 0.405 |
| Urine | All | 0.421 |
|  | HC | 0.436 |
|  | BPH | 0.418 |
|  | PCa | 0.395 |

**Supplementary Table 2.** Summary of clinical studies investigating EVs RNAs as potential PCa biomarkers.

| **Targets** |  | **Body fluids** | **Number of participants** | **EVs isolation methods** | **EVs targets testing methods** | **Diagnostic value/outcome** | **Ref** |
| --- | --- | --- | --- | --- | --- | --- | --- |
| miR-141 | ↑ | Serum | 20 PCa vs 20 BPH vs 20 HC follow-up cohort: 51 PCa (20 mPCa vs 30 LPCa ) | ExoQuick Exosome Precipitation Solution | RT-qPCR | • Higher levels in serum EVs than in whole serum.  • Higher levels in PCa patients vs BPH patients (3.85-fold, P=0.0007) and healthy controls (4.06-fold, P=0.0005).  • EVs miRNA levels associated with PSA≥10, GS≥8, and T3/T4 stages.  • Higher levels in LPCa vs mPCa: AUC=0.8694, sensitivity=80%, specificity=87.1%. | Li, et al. 16 |
|  | ↑ | Serum | 47 recurrent PCa vs 72 non-recurrent PCa | ExoMiR extraction kit | TaqMan RT-qPCR(miR-375 and miR-141) | •miR-141 and miR-375 were associated with recurrent (metastatic) PCa following radical prostatectomy. | Bryant, et al. 12 |
|  | ↑ | Plasma | 78 PCa (55 LPCa, 16 mPCa) vs 28 HC | a filter concentrator with a 150-kDa molecular weight cutoff for MVs extraction, Qiagen miRNeasy for MVs-RNA extraction | Microarray (Profiling of 742 miRNAs)  RT-qPCR validation | • all 78 PCa vs 28 HC : 12 differentially expressed miRNAs, 11 miRNA increased(miR-107, miR-130b, miR-141, miR-2110, miR-301a, miR-326, miR-331-3p, miR-432, miR-484,miR-574-3p, miRr-625), one decreased (miR-181a-2)  • 55 non-metastatic PCa vs 28 HC: 10 differentially expressed miRNAs, 9 miRNA increased(miR-107, miR-141, miR-2110, miR-301a, miR-326, miR-432, miR-484, miR-574-3p, miR-625), one decreased (miR-181a-2) | Bryant, et al. 12 |
|  | ↑ | Urine | 35 PCa vs 35 HC | Lectins, phytohemagglutinin, and concanavalin A induce agglutination of EVs | RT-qPCR | • PCa vs HC :The levels of miR-574-3p,miR-141-5p, and miR-21-5p were significantly up-regulated associated with PCa.  • miR-574-3p: AUC=0.85,miR-141-5p: AUC=0.86, miR-21-5p: AUC=0.65. | Samsonov, et al. 18 |
| miR-375 | ↑ | Serum | 47 recurrent PCa vs 72 non-recurrent PCa | ExoMiR extraction kit | TaqMan RT-qPCR(miR-375 and miR-141) | • miR-141 and miR-375were associated with recurrent (metastatic) PCa following radical prostatectomy. | Bryant, et al.12 |
|  | ↑ | Plasma | Training cohort: 23 CRPC Validation cohort: 100 CRPC | ExoQuick Exosome Precipitation Solution | Training cohort: RNA sequencing  Validation cohort: RT-qPCR(miR-1290, miR-1246, miR-375) | • miRNA levels significantly associated with poor overall survival.  • predictive performance improved via combination of ADT failure time and PSA level at time of CRPC stage with miRNA levels, with AUC increased from 0.660 to 0.730. | Huang, et al. 17 |
|  | ↑ | Plasma | 78 PCa (55 LPCa, 16 mPCa) vs 28 HC | a filter concentrator with a 150-kDa molecular weight cutoff for MVs extraction, Qiagen miRNeasy for MVs-RNA extraction | Microarray (Profiling of 742 miRNAs )  RT-qPCR validation | •16 mPCa vs 55 non-metastatic PCa : 16 differentially expressed miRNAs, 15 miRNA increased (miR-582-3p, miR-20a,miR-375, miR-200b, miR-379, miR-513a-5p, miR-577, miR-23a*, miRr-1236, miR-609, miR-17, miR-619, miR-624*, miR-198, miR-130b), one decreased (miR-572) | Bryant, et al. 12 |
|  | ↑ | Urine | 60 PCa vs 10 HC | differential centrifugation | RT-qPCR (miR-21, miR-141, miR-214, miR-375, let-7c) | • EVs miR-21, miR-375and let-7c were significantly upregulated in PCa vs HC, but no differences were found for miR-141.  • A panel combining miR-21 and miR-375is suggested to distinguish PCa patients and healthy subjects (AUC of 0.872). | Foj, et al. 15 |
|  | ↓ | Urine | Training cohort: 4 HC vs 9 PCa Validation cohort: 26 HC vs 48 PCa | differential (ultra) centrifugation | Training cohort: NGS  Validation cohort: a stemloop RT-PCR | • PCa vs HC: NGS identified top 10 PCa differentially expressed (Log2 fold change > 2) miRNAs in urinary EVs. Higher levels in PCa: miR-10a-5p, miR-204-5p, miR-30a-3p ; Lower levels in PCa: miR-375, miR-21-5p, miR-141-3p, Let-7c-5p, miR-26b-5p, miR-101-3p, Let-7b-5p.  • The diagnostic performance of three isomiRs combination of miR21, miR375and miR204 resulted in an AUC of 0.866, compared to PSA AUC of 0.707 and the corresponding three mature miRNAs AUC of 0.766. | Koppers-Lalic, et al. 20 |
| PCA3 lncRNA ERG mRNA | ↑ | Urine | 106 controls ( Bx Neg) vs 89 PCa ( Bx Pos) | Urine Clinical Sample Concentrator Kit (Exosome Diagnostics) | RT-qPCR computed EXO106 score (the sum of normalized PCA3 and ERG RNA levels) | • EXO106 score demonstrated good clinical performance in predicting biopsy result for both any PCa (AUC=0.715) and high-grade PCa (AUC=0.764).  • The clinical performance was improved with a combination of EXO106 and SOC (standard of care= PSA, age, race or family history) (any PCa: AUC=0.715; high-grade PCa: AUC=0.803). | Donovan, et al. 19 |
|  | ↑ | Urine | Training cohort: 255 patients with PSA(2 to 20 ng/mL)and biopsy outcomes Validation cohort: 519 patients with PSA(2 to 20 ng/mL)and prognostic score | Urine Clinical Sample Concentrator Kit | urine exosome gene expression assay (the ExoDx Prostate IntelliScore urine exosome assay): RT-qPCR | • Training cohort: Gene Expression Assay in combination with SOC (AUC=0.77) significantly improved performance SOC alone (AUC=0.66) for predicting high-grade PCa (HG PCa, GS ≥ 7) from low-grade PCa (GS = 6) and benign disease.  • Validation cohort: Compared with the PCPTRC (AUC=0.62) and PSA alone (AUC=0.55), Gene Expression Assay (AUC=0.71) demonstrated improved performance.  • Using a predefined cut point, 27% biopsies would have been avoided, missing only 5% of patients with dominant pattern 4 high-risk GS7 disease. | McKiernan, et al. 21 |
|  | ↑ | Urine (after DRE) | 15 PCa (Bx Pos) vs 14 controls (Bx Neg) | filtration through a 100 kDa filter | RT-qPCR | • The biomarker levels were highest in whole urine and significantly higher after DRE in all substrates (whole urine, cell pellet and EVs).  • In the EVs substrate of urine, no significant differences were found inPCA3,ERG, KLK3 between Bx Pos and Bx Neg PCa patients. | Hendriks, et al. 13 |
|  | ↑ | Urine (after DRE) | 12 HC vs 14 GS=6 PCa vs 26 GS≥7 PCa | Ultrafiltration | TaqMan qPCR | • EVs RNA were richer than RNA from cell pellets in urine.  • PCa vs HC: In comparison to HC, both of the GS6 and GS7+ PCa groups had significantly higher expression of PCA3, while only the GS7+ group had significantly higher expression of ERG(no difference between the two GS groups).  • No significant difference in KLK3expression between any of the groups. | Pellegrini, et al. 14 |
| TMPRSS2:ERG mRNA | ↑ | Urine (urine collected prior to RP) | 21 PCa: urinary EVs vs corresponding prostatectomy tissue from the same patients 39 PCa (Bx Pos) vs 47 controls (Bx Neg) | Filtration: a 100k MWCO filtration concentrator (Millipore) | RT-qPCR (mRNA of TMPRSS2:ERG, BIRC5,ERG, PCA3 and TMPRSS2) | • urinary EVs had a sensitivity: 81% (13/16), specificity: 80% (4/5) and an overall accuracy: 81% (17/21) for non-invasive detection of TMPRSS2:ERG vs RP tissue.  • The rate of TMPRSS2:ERG exoRNA detection was found to increase with age and the expression level correlated with Bx Pos status.  • TMPRSS2:ERG: AUC=0.744, AR: AUC=0.558, BIRC5: AUC=0.674, ERG: AUC=0.785,PCA3: AUC=0.681. | Motamedinia, et al. 22 |
| miR-21 | ↑ | Urine | 60 PCa vs 10 HC | differential centrifugation | RT-qPCR (miR-21, miR-141, miR-214, miR-375, let-7c) | • EVs miR-21, miR-375 and let-7c were significantly upregulated in PCa vs HC, but no differences were found for miR-141.  • A panel combining miR-21 and miR-375 is suggested to distinguish PCa patients and healthy subjects (AUC of 0.872). | Foj, et al. 15 |
|  | ↑ | Urine | 35 PCa vs 35 HC | Lectins, phytohemagglutinin, and concanavalin A induce agglutination of EVs | RT-qPCR | • PCa vs HC :The levels of miR-574-3p,miR-141-5p, and miR-21-5pwere significantly up-regulated associated with PCa.  • miR-574-3p: AUC=0.85, miR-141-5p: AUC=0.86, miR-21-5p: AUC=0.65. | Samsonov, et al. 18 |
|  | ↓ | Urine | Training cohort: 4 HC vs 9 PCa Validation cohort: 26 HC vs 48 PCa | differential (ultra) centrifugation | Training cohort: NGS  Validation cohort: a stemloop RT-PCR | • PCa vs HC: NGS identified top 10 PCa differentially expressed (Log2 fold change > 2) miRNAs in urinary EVs. Higher levels in PCa: miR-10a-5p, miR-204-5p, miR-30a-3p ; Lower levels in PCa: miR-375, miR-21-5p, miR-141-3p, Let-7c-5p, miR-26b-5p, miR-101-3p, Let-7b-5p.  • The diagnostic performance of three isomiRs combination of miR21, miR375 and miR204 resulted in an AUC of 0.866, compared to PSA AUC of 0.707 and the corresponding three mature miRNAs AUC of 0.766. | Koppers-Lalic, et al. 20 |
| Let-7c | ↑ | Urine | 60 PCa vs 10 HC | differential centrifugation | RT-qPCR (miR-21, miR-141, miR-214, miR-375, let-7c) | • EVs miR-21, miR-375 and let-7c were significantly upregulated in PCa vs HC, but no differences were found for miR-141.  •let-7c was significantly correlated with PCa clinical stage. | Foj, et al. 15 |
|  | ↓ | Urine | Training cohort: 4 HC vs 9 PCa Validation cohort: 26 HC vs 48 PCa | differential (ultra) centrifugation | Training cohort: NGS  Validation cohort: a stemloop RT-PCR | • PCa vs HC: NGS identified top 10 PCa differentially expressed (Log2 fold change > 2) miRNAs in urinary EVs. Higher levels in PCa: miR-10a-5p, miR-204-5p, miR-30a-3p; Lower levels in PCa: miR-375, miR-21-5p, miR-141-3p, Let-7c-5p, miR-26b-5p, miR-101-3p, Let-7b-5p. | Koppers-Lalic, et al. 20 |

Note:↑/↓: The expression was increased/decreased in PCa.

# Abbreviations：HC, healthy controls; BPH, benign prostate hyperplasia; PCa, prostate cancer; PSA, prostate-specific antigen; mPCa, metastatic PCa; LPCa, localized PCa; CRPC, castration-resistant prostate cancer; Bx Pos, biopsy positive; Bx Neg, biopsy negative; DRE, digital rectal examination; NGS, next generation sequencing; PCPTRC, Prostate Cancer Prevention Trial Risk Calculator; ADT, androgen-deprivation therapy.
